# Label-Free Bioanalyte Detection from Nanometer to Micrometer Dimensions—Molecular Imprinting and QCMs †

**DOI:** 10.3390/bios8020052

**Published:** 2018-06-01

**Authors:** Adnan Mujahid, Ghulam Mustafa, Franz L. Dickert

**Affiliations:** 1 Department of Analytical Chemistry, University of Vienna, Währinger Straße 38, A-1090 Vienna, Austria; adnanmujahid.chem@pu.edu.pk; 2 Institute of Chemistry, University of the Punjab, Quaid-i-Azam Campus, Lahore 54590, Pakistan; 3 Center for Interdisciplinary Research in Basic Sciences, International Islamic University, H-10, Islamabad 44000, Pakistan; gmustafa@iiu.edu.pk

**Keywords:** molecular imprinting, quartz crystal microbalance (QCM), bioanalytes, label-free sensors, bacteria, viruses

## Abstract

Modern diagnostic tools and immunoassay protocols urges direct analyte recognition based on its intrinsic behavior without using any labeling indicator. This not only improves the detection reliability, but also reduces sample preparation time and complexity involved during labeling step. Label-free biosensor devices are capable of monitoring analyte physiochemical properties such as binding sensitivity and selectivity, affinity constants and other dynamics of molecular recognition. The interface of a typical biosensor could range from natural antibodies to synthetic receptors for example molecular imprinted polymers (MIPs). The foremost advantages of using MIPs are their high binding selectivity comparable to natural antibodies, straightforward synthesis in short time, high thermal/chemical stability and compatibility with different transducers. Quartz crystal microbalance (QCM) resonators are leading acoustic devices that are extensively used for mass-sensitive measurements. Highlight features of QCM devices include low cost fabrication, room temperature operation, and most importantly ability to monitor extremely low mass shifts, thus potentially a universal transducer. The combination of MIPs with quartz QCM has turned out as a prominent sensing system for label-free recognition of diverse bioanalytes. In this article, we shall encompass the potential applications of MIP-QCM sensors exclusively label-free recognition of bacteria and virus species as representative micro and nanosized bioanalytes.

## 1. Introduction

Modern day analytical tools are considered to offer highly sensitive and selective response, short analysis time with straightforward operation, large sample throughput, and miniaturized design with improved reliability. In addition to this, the price of such analytical machines should be less for common use. Nonetheless, large instrumentation, extensive and laborious sample preparation steps and need of trained personnel to interpret analytical data are some of the concerns of traditional analytical instruments. Alternatively, biosensors [1,2,3] are smart miniaturized devices that can precisely quantify the target analytes in complex mixtures and transform this information into measurable signal. The interfacial coatings of such devices are typical biologically derived materials such as antibodies [4,5], enzymes [6,7] and others. These receptors are potentially capable of monitoring the target analyte among competing or related species. Such high selectivity is attributed to special recognition properties of biological receptors.

Since all the receptor cells in living organisms contribute to their functionalities in predefined order and show precise interactions for target analytes in a highly selective and specific way. As a result, the response to other competing molecules is close to negligible. This sort of discrimination for target molecules among closely related species lays the foundation of molecular recognition [8,9]. Biosensor devices are comprised of such a highly specific biological receptor interface that selectively binds with targets. In order to enhance this binding and also to convert this information into a measurable electronic, optical, acoustic or any other signal, some labeling molecules [10,11,12] for example proteins and quantum dots are used. Although the main purpose of using labels is to improve the binding interactions and transform this information for transduction, it could, however, lead to more complexity. This includes change in analyte recognition dynamics—for example, the non-specific bindings of labeling molecule may lead to false positive or negatives during simple screening tests. This somewhat limits the reliability of analytical tests, and, furthermore, analysis time and cost are also increased. The recognition of target analytes based on its intrinsic physiochemical features is the basis of label-free detection protocols [13]. The foremost advantages of this strategy are improving analytical reliability due to direct target receptor binding, reducing the sample complexity due to the absence of non-specific interactions of labeling indicator and ultimately managing all this at a low-cost by avoiding the labeling step. Therefore, label-free biosensors [14,15,16] found a variety of applications in diverse fields including clinical diagnostics, bioanalyte recognition, environmental analysis and many others.

The interfacial part of a typical label-free biosensor may contain biologically derived receptors such as natural antibodies, proteins, enzymes and even viruses as well. Recently, Poghossian et al. [17] reported an interesting strategy of using virus nanoparticles as scaffolds for immobilization of penicillinase enzyme. They integrated this virus–enzyme hybrid receptor material with a field effect device i.e., capacitive sensor for detecting penicillin in bovine milk. Apart from high sensitivity for penicillin and low detection limit down to 50 µM, the developed sensor exhibited surprisingly good long-term stability as the sensor performance remains the same even after one year of repeated use.

Although natural receptors show high binding affinity and selectivity, their synthetic counterparts offer more flexibility in terms of preparation/derivation protocols, robust in harsh conditions, adaptable with a transducer device and ultimately their price is much reduced. This triggers more research in developing synthetic receptors [18,19,20] for biosensing applications and, consequently, a number of potential strategies appeared in the literature. Among many, molecular imprinting [21,22,23,24] emerged as a promising choice for synthesizing highly adaptable sensor coatings. During the last decade, molecular imprinted materials attracted considerable attention because of their high selectivity as good as natural antibodies [25]. Apart from their high binding affinity and selectivity for targets in complex mixtures, their synthesis is uncomplicated and is completed in a short time. They are robust and stable in corrosive environments. These features make molecular imprinted materials the first preference for biosensor coatings [26] and can be combined with diverse transducers [27,28,29,30].

The selectivity is mainly driven by receptor material while sensitivity is controlled by the type of transducer. The transduction principle, robustness, and miniaturization for field measurements and ability for wireless integration [31] are the essential aspects for an efficient label-free biosensor. With all these features, acoustic resonators such as quartz crystal microbalance (QCM) [32,33] are considered as suitable gravimetric devices that have been comprehensively used as mass-sensitive sensors [34,35]. QCMs having certain chemical coatings can quantify extremely small mass changes i.e., as low as down to picograms [36]. Therefore, they are often regarded as smart tiny balances. Furthermore, QCM devices are known as universal transducers since mass is a fundamental property of any target molecule and can be determined by such sensors. This means that, if a target analyte does not possess any substantial electrical, optical, thermal, magnetic or any other property, it still can be sensed by QCM devices. This feature makes QCM devices a top priority in modern sensor design and development.

The combination of MIPs (as synthetic receptors) with QCM devices (as transducers) has turned out to be highly prolific label-free sensors [37,38,39], which are used for a wide variety of applications. This covers the detection of clinical biomarkers [40,41], pharmaceutical drugs [42], environmental toxins [43,44], and especially diverse bioanalytes species including viruses [45], bacteria [46], proteins [47] and others [48]. The outstanding recognition properties of imprinted polymeric receptors and efficient gravimetric transduction by QCM allow the detection of target analytes at trace concentrations in real-time samples. In this work, we shall describe the selected bioanalytes sensing examples using MIP-QCM devices with the most relevant literature. The article is divided into three parts: the first part deals with diverse molecular imprinting strategies [49,50] for artificial receptor design, the second part will briefly highlight QCM operation and fabrication as a gravimetric transducer, and, finally, in the third section, the key applications for label-free detection of bacteria and viruses will be covered. In the conclusions section, the prime advantages of MIP-QCM sensors are discussed and compared with established technologies. Furthermore, the potential future applications are also described.

## 2. Biomimetic Recognition via Molecular Imprinting

Molecular imprinting is a modern method of synthesizing biomimetic receptors that can be used for the detection of diverse bioanalytes. The success of molecular imprinting in designing synthetic recognition materials is largely credited to pioneering work of Guenter Wulff and Klaus Mosbach. The research group of Wulff showed that the catalytic activities demonstrated by covalently molecular imprinted material were as good as enzymes [51,52]. Mosbach [53,54,55] utilized the idea of non-covalent interactions to generate the receptor sites in imprinted polymers.

Imprinted polymers are the artificial recognition materials that possess high selectivity for the target molecules. The binding between MIP and analyte is analogous to the lock and key principle as observed in many natural phenomena. Since MIPs are highly proficient for molecular recognition, they can recognize target analytes based on molecular structure or chemical functionality rather than geometrical size based fitting in imprinted cavities. Thus, MIPs can distinguish between two analytes having exactly the same geometrical dimensions but different molecular structures, which suggest that MIPs offer complementary chemical fits to target analytes. MIPs can be designed for a wide range of targets including bacteria [56], yeast cells [57], red blood cells [58,59], viruses [60], proteins [61], enzymes [62] and other bioanalytes [63] for a number of technological applications [64,65]. This includes the precise and reliable clinical diagnostics [66,67,68], efficient separations [69,70,71], drug delivery [72,73,74], and smart chemical sensor devices [75]. The resulting perspectives in sensor technologies are immense due to versatile applications of MIPs by combining with suitable transducers.

Several parameters like nature of analyte, type of solvent used for polymerization, functional monomers, cross-linking agents and initiators, etc. can influence performance of the final imprinted polymer. The type of imprinting is also of significant importance i.e., covalent, non-covalent, semi-covalent or others, and, furthermore, the decisive factor is the selection of a suitable imprinting technique for a specific class of analytes. In the coming sub-headings, we shall briefly discuss the type of imprinting [76,77], i.e., non-covalent approach for bioanalytes recognition as well as how to execute the imprinting process of bioanalytes to achieve highly responsive and selective sensor interfaces.

### 2.1. Non-Covalent Imprinting a Biomimetic Approach

In living organisms, all of the intercellular and intracellular communications are generally carried out through non-covalent interactions e.g., binding of antibodies with antigens, interaction of receptors with hormones, enzymatic catalysis and others. The rich diversity of the non-covalent interactions [78] in nature stimulated the scientists to fabricate the artificial receptors using molecular imprinting technology [79]. Monomer molecules bearing functional groups complementary to that of template leads to the development of a pre-polymer complex based on non-covalent interactions. With the addition of a suitable cross-linker, this mixture is polymerized to rigidly fix the spatial arrangement of oligomer chains. The extraction of template molecules from the polymer does not involve any bond cleavage, and, after its exclusion, the cavities of defined size, shape and chemical functionality corresponding to the template are formed. These cavities are highly selective and can bind reversibly the target molecules offering matching geometrical and chemical fittings. The affinity between template molecules and imprinted cavities can be recognized as donor–acceptor interactions, hydrogen bonding, dipole–dipole attraction, van der Waals forces, and π–π interactions. Since the focus of label-free recognition is to detect target based on its intrinsic properties therefore, non-covalent imprinting is the most appropriate choice to meet this requirement.

### 2.2. Process of Bioanalyte Imprinting

The process of bioanalyte imprinting is of utmost importance in the development of efficient sensor receptor coatings. Since there are several methods of imprinting, the main objective of label-free detection is to analyze targets based on its fundamental properties without using any external agent. Thus, a typical imprinted layer should recognize targets as such, and, furthermore, the synthesized polymer interface may offer completely reversible interaction sites, faster diffusion pathways and complementary geometrical and chemical fitting to target analyte. In order to design such imprinted surface, the target bioanalyte for example bacterial cells can be directly used as structural templates to develop adapted cavities on polymer surface. This was first demonstrated by group of Vulfson [80,81] where they synthesized bacteria-imprinted polymer beads in an organic-aqueous environment. By knowing the fact that bacterial cells can partition themselves between organic and aqueous phase, the authors chemically reacted hydrophilic polyamine and diacid chloride in the organic phase along with bacterial suspension. The diacid chloride can form a covalent linkage with a bacterial cell wall and also with polyamines, thus resulting in the formation of polyamides while keeping bacterial cells intact at the surface. The interface of polymer beads was modified with perfluoropolyether to block residual amino groups, and, later, bacterial cells were removed from the polymer surface by acid hydrolysis. The developed polyamide beads contain spatially functionalized adapted patches/cavities at the surface, which were identical to template bacterial cells and thus can be used for selective recognition. The authors named this strategy “*lithographic print*” of microorganisms, which could be used for precisely reproducing the template cell structure at the polymer interface.

**Figure 1:**
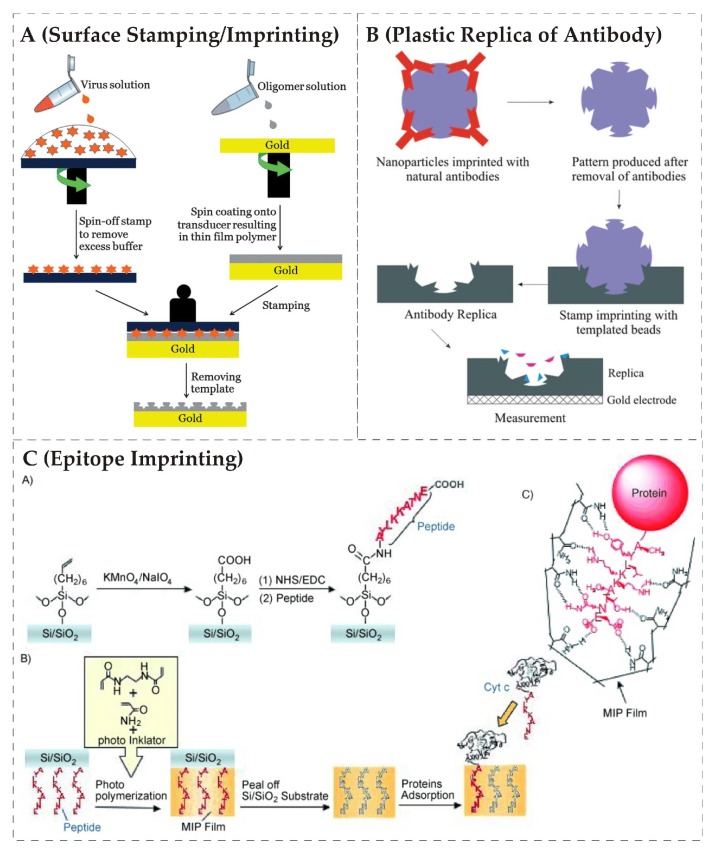
(**A**) *surface stamping/imprinting* method where the template i.e., viruses are assembled on stamp and then pressed over pre-polymer layer to generate patterned surface, reproduced with permission from [88]; (**B**) synthesis of *plastic replica of antibodies* is shown, in the first step antibody templates are imprinted in nanoparticles; afterwards, the templates are removed and resulting nanoparticles are collected on a stamp for pressing the pre-polymer layer, reproduced with permission from [83]; (**C**) *epitope imprinting*; in the first step, the glass surface was modified; the second stage shows the attachment of the peptide and subsequent molecular imprinted polymer MIP formation; then, the glass substrate is removed, and, finally, the attachment to protein through its epitope is shown, reproduced with permission from [87].

*Surface stamping/imprinting* is a soft lithographic technique [82] that has extensively followed the route for imprinting of proteins and microorganisms including whole biological cells as well. In this method, the template/target analyte units are closely assembled on a suitable substrate to have high imprinting density on the polymer surface. The template stamp is pressed over a pre-polymer (uncured) layer i.e., already coated on the transducer electrode. In this way, all the geometrical and structural details are transferred on the polymer surface. After curing of polymer layers for a specific period of time and under a controlled environment, the template units are removed, whereas, at the surface, well-defined cavities are produced in this process, which can selectively and reversibly accommodate target analyte. A typical illustration of surface stamping/lithographic technique is shown in the following Figure 1A. This is also called surface molecular imprinting and it can be applied to imprint larger biological cells such as bacteria, yeast, red blood cells having sizes in micrometer range and also to viruses as well having nanometer dimensions.

A further advancement to this process [83] is the production of *plastic replica of antibodies* using natural antibodies as template for preparing MIP nanoparticles, which are then used as stamps to transfer a pattern on the pre-polymer surface. This is a two-step process as in the first stage; the antibodies-imprinted nanoparticles can be synthesized by taking a monomer solution along with natural immunoglobulin (Ig) and then precipitated in a suitable solvent. In the second step, antibody-imprinted nanoparticles are assembled on a glass slide to from a stamp that is pressed over pre-polymer surface for transferring antibodies impression on polymer layer interface, thus generating precise *plastic replica of antibodies* on a polymer surface. In this strategy, complete details of antibodies are transferred to synthetic polymer surface, which yields suitable plastic copies of template natural antibodies. This strategy has two key advantages: first, is the high selectivity for target analyte recognition, which is typical of the natural antibody binding property; secondly, the availability of a higher surface area due to a nanoparticle stamp used for imprinting, as it would lead to generating a larger number of binding sites, thus leading to enhanced sensitivity. Additionally, the interaction could be completely reversible due to low energy between layer and particle. A schematic representation of this strategy is shown in Figure 1B.

*Epitope imprinting* is another method of choice for dealing larger bioanalytes such as proteins and peptides. In this method, a specific short peptide sequence (usually the terminal part of target protein) is taken as a template to generate recognition sites in MIPs. After removal of this template, the resulting imprinted sites can recognize the whole protein structure through its epitope i.e., used during the imprinting process. Rachkov and Minoura [84,85] termed this method the epitope approach and explained that the method is inspired from nature, where the antibody recognizes antigens through interacting with only a small part of it, i.e., epitope of antigen. Thus, unlike imprinting the whole biological cell structure, this method provides an alternate method by taking a suitable peptide sequence [86] as a template. This peptide sequence is accommodated in the imprinted site during protein recognition. It is important to mention here that the nature of the peptide sequence [87] plays an essential role in achieving enhanced recognition properties for target protein structure. Epitope imprinting provides the opportunity to capture proteins in the native environment. In view of sensor coatings, this method has been widely used for recognition of virus proteins. A schematic representation of epitope imprinting has been displayed in Figure 1C.

## 3. QCM Devices for Label-Free Transduction

Focusing on label-free sensing, the target analyte needs to be detected based on its inherent properties, and, in this perspective, mass is a fundamental feature of every target molecule that can be sensed by smart, miniaturized balances such as QCM. They are regarded as acoustic/gravimetric devices that are extensively used as mass-sensitive transducers. The fundamental principle of QCM transduction is based on piezoelectricity when applying voltage to such materials, mechanical deformation takes place, making them resonate at a certain frequency. When some mass is deposited on such materials, the oscillation frequency is dropped depending on loaded mass. Thus, the *change in frequency* is taken as *change in mass adsorbed* on the QCM surface, which leads to the development of mass-sensitive sensors. Sauerbrey [89] mathematically derived the relationship between frequency shifts and mass loadings as shown in Equation (1):

(1) $$\frac{\Delta f}{f_{o}}=-\frac{\Delta m}{m}$$

In this equation, ∆*f* is the shift in frequency on loading of mass ∆*m*, while *f_o_* is the fundamental resonating frequency and *m* is the mass of unloaded resonator. The change in frequency depends on a number of different factors including fundamental resonating frequency, the amount of mass loading, material constants such as density and shear modulus of quartz wafer. However, while dealing with bioanalyte detection, one side of the QCM devices is in contact with liquid phase; therefore, the viscosity of the liquid medium also needs to be considered to understand sensitivity, noise and resolution issues [90]. Therefore, the relationship is modified as proposed by Kanazawa and Gordon [91] and shown as follows in Equation (2):

(2) $$\Delta f=f_{o}^{3/2}{\left(\frac{\eta \rho }{\pi \mu_{q}\rho_{q}}\right)}^{1/2}$$

In this equation, *η* and *ρ* are viscosity and density of in contact liquid medium, respectively, whereas *ρ_q_* represents density of quartz i.e., 2.648 g/cm^3^ and *µ_q_* is shear modulus of quartz having a value 2.947 × 10^11^ g/cms^2^. In typical QCM design, AT-cut quartz is used i.e., singly rotated Y-cut quartz plate having a normal axis parallel to the *y*-axis with θ ≅ 35.25° [92]. The advantages of AT-cut quartz are its high thermal stability against a wide range of temperatures and low fabrication cost. For sensor fabrication, inert metals such as gold electrodes are printed on both sides of quartz, which are connected with an oscillator circuit. On applying voltage across the electrodes, mechanical deformation takes place in quartz crystal, which makes QCM devices resonate. Since every analyte has mass and its deposition on QCM electrode would result in frequency shifts; therefore, in order to make QCM a selective sensor device, the electrode surface needs to be covered with highly specific receptor coatings. Furthermore, larger electrode geometry could improve sensitivity as well since it offers larger receptor interfacial layer coatings, which would result in more interaction sites for analyte recognition.

The fundamental resonance frequency has a substantial importance on sensitivity of QCM sensors [93]. From the above relationship, it can be seen that the frequency shift increases with increasing the resonating frequency of the devices. The usual operating frequency of QCM lies in the range of 5–20 MHz; however, devices having a higher fundamental frequency up to 100 MHz [94,95] and above [96] are also reported in the literature for biosensing applications. Such high frequency QCM sensors offer much improved detection limits. It is also important to mention here that increased fundamental resonance frequency demands more thin quartz wafer, therefore going above 20 MHz make QCM devices more fragile and difficult to handle especially in liquid phase. The alternate approach is to work at higher odd harmonic modes [97] of low fundamental frequency QCM devices; however, the noise is also increased considerably.

Recently, QCM devices combined with an ionic liquid–polymer composite layer were reported [98] for chemical sensing as well as molecular weight estimation of diverse analyte vapors. Warner and coworkers [99] used QCM with dissipation (QCM-D) coated with 1-hexyl-3-methylimidazoliumbis (trifluoromethylsulfonyl)imide ([HMIm][NTf_2_]) combined polymethylmethacrylate (PMMA) for discriminating different alcohols. They tested methanol, ethanol, 1-propanol, 2-propanol, 1-butanol, 2-butanol, 3-methyl-1-butanol and 1-hexanol. The authors introduced the virtual sensor array (VSA) concept [100], which suggests that, when a single device coated with a chemical sensitive layer is measured at multiple harmonics, it yields a series of independent sensor signals. In addition, measuring the sensor layer having a fixed thickness in multiple harmonics is equivalent to measuring multiple layer thicknesses at single harmonics. Therefore, measuring a single device with a fixed layer height at multiple harmonics generates a series of sensor signals that can be used for discriminating distinct and closely related analytes. It was demonstrated that the developed QCM VSA can be successfully applied for precise recognition of above-mentioned alcohols and also their molecular weight approximation, thus offering a two-dimensional analysis platform. In another study [101], this approach was extended for monitoring adulteration in petroleum fuels and also discriminating different gasoline grades.

**Figure 2:**
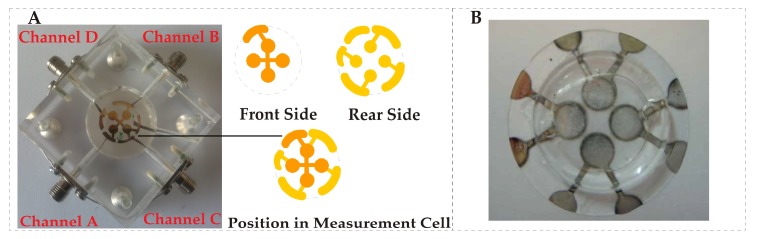
(**A**) shows a tetra electrode quartz crystal microbalance QCM design and its placement in plexiglass measuring cell, reproduced with permission from [102]; (**B**) another deign of tetra electrode QCM placed in polydimethylsiloxane (PDMS) chamber, reproduced with permission from [103].

For developing a QCM sensor array, multiple electrode geometries can be printed on a single QCM sheet such as dual, tri or even tetra electrodes [102,103]. The multi-electrodes can be covered with different receptor layers, allowing for sensing of multiple targets on a single QCM wafer. Furthermore, in order to evaluate non-specific interactions and compensate for unwanted frequency shifts, one electrode channel may be taken as a reference, which could improve the reliability of measurements. However, while developing more than one electrode on a single QCM wafer, there should be a suitable distance between the electrodes to avoid the cross-talk. Figure 2A represents a multichannel tetra electrode QCM sensor array placed in a plexiglass measuring cell. This array can be used for simultaneous measurements of three diverse analytes using three different receptors covered on three electrodes, while the fourth channel could be taken as a reference. Figure 2B shows another design of a tetra electrode QCM sensor array. In general, QCM sensors are efficient label-free gravimetric transducers that are extremely sensitive to small mass shifts as low as to the picogram level [104]. Apart from exceptionally high sensitivity, QCM devices are robust and stable against temperature shifts, possess low fabrication costs and offer rapid response on mass loading.

## 4. MIP-QCM Sensors for Bioanalytes

There is a diverse variety of bioanalytes that can be recognized using a MIP-QCM setup; however, here we shall focus on the detection of bacteria and viruses as target analytes having sizes from micrometer to nanometer range, respectively. The exemplary applications of MIP-QCM sensors for label-free recognition of such bioanalytes are discussed as follows.

### 4.1. Detection of Bacterial Species

**Figure 3:**
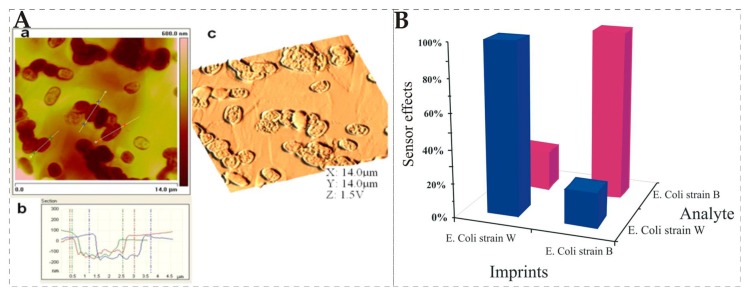
(**A**): (**a**) shows polyurethane layer imprinted with the artificial stamp of *E. coli*; (**b**) sectional view for accessing depth of imprinted cavities; (**c**) 3D image of polyurethane layer; (**B**) presents cross-sensitivity of two different sensor layers imprinted with *E. coli* B and W strains, respectively, it is clear that both layers showed higher sensor effects to *E. coli* strain, which was used as a template for imprinting and minimal response to non-target strain, adapted from [105].

Label-free detection of pathogenic microorganisms has a significant importance as it could poison food, drinking water and others, thus causing serious health problems for humans. Modern analytical methods focused on their quick identification and precise quantification in complex mixtures. MIP-QCM sensors have shown considerable success for detection of different *Escherichia coli* (*E. coli*) strains. A surface lithographic stamping method is extensively followed for imprinting of *E. coli* in several research articles. In this method, *E. coli* cells of suitable concentration are assembled on a glass slide to use as a *master stamp* that is pressed over an already spin-coated pre-polymer e.g., polyurethane layer with the help of clamps. During the curing of polyurethane layer, the structural details are transferred to a polymer layer and afterwards the stamp is removed. The temple is washed out from a polymer and the resultant surface contains the adapted cavities of *E. coli*, which can recognize the specific *E. coli* strain that was used during imprinting. A further advancement to this strategy is the use of an *artificial stamp* [105,106] of *E. coli* that can be produced by simply pressing an un-cured silicon polymer over an already imprinted polyurethane surface. After hardening of a silicon polymer i.e., PDMS, it is taken off and can be used as a replica stamp of *E. coli*. The primary advantage of using the artificial stamp is that there is no need for actual *E. coli* cells every time for surface stamping. Figure 3A showed an atomic force microscope (AFM) image of a polymer surface that was patterned by such an artificial stamp. The developed cavities are highly selective in recognizing the target *E. coli* strain. For instance, QCM measurements as depicted in Figure 3B showed the relative sensor responses of two different bacterial sensors where one sensor layer was imprinted with an *E. coli* W strain and the other was with a *E. coli* B strain. Sensor surface imprinted with *E. coli* W stamps showed the highest sensor response for *E. coli* W strain than the *E. coli* B strain. Furthermore, the same trend was observed when a polymer surface was stamped with *E. coli* B strain. This demonstrates the high selectivity developed by the surface stamping technique.

For improving sensitivity, Zhang et al. [107] developed hierarchal patterned imprinted polystyrene structures for improved attachment of *E. coli* cells for obtaining higher frequency shifts. Schnettelker et al. [108] developed a modified method of surface imprinting where *E. coli* cells were first immobilized on the transducer surface and then in-situ polymerization was followed. The bacterial cells were attached with a QCM electrode using a suitable linker and then a polymer layer of controlled thickness was developed on this surface. The removal of template cells leaves behind adapted cavities. The critical factor of this strategy is the control over polymer layer height to have complete structural details of cells to be imprinted. Since a thinner layer would not be enough to entirely interact with template cells, whereas a thicker polymer layer would completely bury the cells and would not allow them to escape. The authors proposed that thickness of the polymer layer can be controlled by making a suitable dilution of oligomer solution.

Apart from developing different surface imprinting strategies, sensitivity can also be improved by selecting suitable polymer material. For instance, Spieker and Lieberzeit [109] used different polymers for imprinting *Bacillus cereus* i.e., gram positive bacteria and compared their sensor effects. The authors tested polyacrylamide, polyurethane, polyvinyl pyrrolidone, polyacrylate and polystyrene. They observed that polyacrylamide showed the highest sensor effect, whereas polyurethane was the second best for enhanced sensor effects.

**Figure 4:**
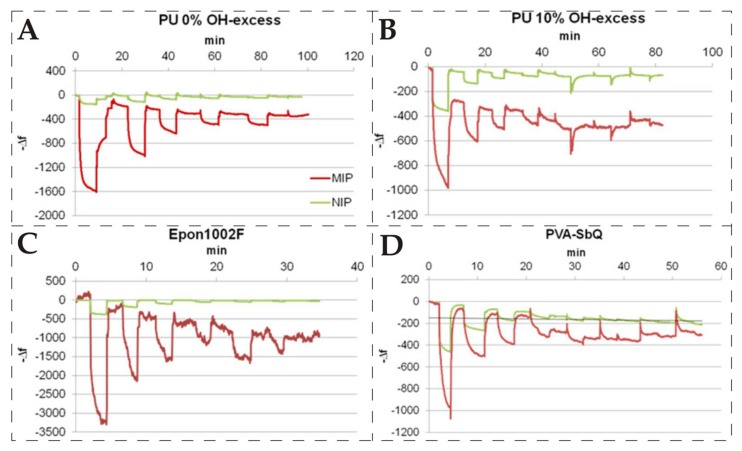
QCM sensor responses of four different MIP layers imprinted with same strains of *E. coli*; (**A**) shows the sensor response of polyurethane layer (0% OH-excess group); (**B**) polyurethane layer (10% OH-excess) group; (**C**) represents sensor effects for Epon 1002F and finally (**D**) shows the response for PVA-SbQ. All sensor layers were exposed to same series of different *E. coli* concentrations i.e., 7.3, 3.6, 1.8, 0.9, 0.7, and 0.4 × 10^7^ CFU/mL, reproduced with permission from [110].

In another report, Poller et al. [110] studied the effect of ready-to-use and ab initio synthesized polymers on sensitivity of *E. coli* detection. The two ready-to-use polymers were Epon 1002F i.e., (derived from liquid epoxy resin and bisphenol A) and Poly(vinyl alcohol)/*N*-methyl-4(4′-formylstyryl)pyridinium methosulfate acetal (PVA-SbQ). The two ab initio synthesized polymers were polyurethanes having 0% OH and 10% OH groups, respectively. All four of the polymers were processed following the surface stamping technique under the same conditions. Each surface imprinted polymer along with corresponding non-imprinted polymer was coated on dual electrode 10 MHz QCM and exposed to a range of *E. coli* concentrations. The comparison of sensor responses of all four devices is shown in Figure 4. This graph clearly indicates that Epon 1002F-MIP showed the highest sensor effect, whereas polyurethane-MIP having 0% OH groups exhibited nearly half of this response. The signal of their corresponding non-imprinted polymers was negligible. The other MIP layers i.e., polyurethane with 10% OH groups and ready-to-use polymer PVA-SbQ showed comparatively much reduced frequency shifts. Furthermore, their corresponding non-imprinted layers exhibited high non-specific binding for *E. coli*. The authors proposed that the high sensitivity of Epon 1002F and polyurethane (0% OH) is due to their stronger electrostatic interactions with *E. coli* cells. Since both of these polymers are negatively charged, whereas, in aqueous solution, *E. coli* cells are surrounded by a cationic layer, thus leading to strong attraction between them. These findings suggest that, apart from imprinting methodology, the nature of the polymer is also important in achieving high sensitivity, and, furthermore, ready-to-use polymers could be a suitable choice to overall reduce the time and effort.

Rapid analysis of bacterial species in complex samples by QCM sensors is advantageous over other transducers, as it offers shorter response time and improved detection limits. Yilmaz et al. [111] investigated the response of MIP layers for *E. coli* sensing using QCM and surface plasmon resonance (SPR) devices. The two sensors, i.e., QCM and SPR, were fabricated with the same polymer material and underwent the same imprinting method. The performance of two sensor devices was compared in Table 1, which revealed that the QCM sensor offers rapid response time of 56 s than SPR having 113 s. The overall time of adsorption, equilibrium and desorption for QCM is about 7 min, while, for SPR, it takes 20 min to complete the cycle. The authors proposed that this could be due to the difference in flow rates of QCM and SPR sensors i.e., 350 μL/min and 150 μL/min, respectively. They studied different isotherms and found that the Langmuir model fit best to experimental data of both sensors having R^2^ for QCM 0.9931 and for SPR 0.9461. The detection limit by QCM devices was calculated to be 3.72 × 10^5^ CFU/mL, which is an order of magnitude lower than SPR i.e., 1.54 × 10^6^ CFU/mL. This indicates that sensitivity of the QCM sensor is appreciably higher than SPR with the same recognition interface. The selectivity of both of the sensors was satisfactorily high for target recognition of *E. coli* comparing to other bacterial species i.e., *Bacillus* and *Streptococcusas*. Furthermore, they can be applied for monitoring *E. coli* concentrations in real samples.

Liu et al. [112] developed a MIP-QCM sensor to monitor staphylococcal enterotoxins (SE) in food samples, which are produced by *Staphylococcus aureus* i.e., a gram positive bacteria. The authors first modified the QCM gold electrode surface with thiols and then a mixture of template cells along with pre-polymer sol-gel was spin coated. After drying the layer, the surface was rinsed with water to remove a physically adsorbed polymer. This layer deposition process was repeated from 3 to 5 times to have a significant mass of polymer layer. The authors imprinted staphylococcal enterotoxins A (SEA) and staphylococcal enterotoxins B (SEB) template cells on 5 MHz QCM-D modified gold electrodes. The QCM-D setup is used here to measure frequency shifts as well as dissipation shifts on analyte interaction with a sensor layer. The imprinting strategy was similar to their previous report [113]; however, the authors reported lower detection limits here using QCM-D and were able to perform multiplex measurements. The developed sensors showed a good recognition response for SEA and SEB cells detection in the range of 0.1–1000 ng/mL. The selectivity of these QCM sensors was tested by exposing them against structurally related SE cells i.e., SEC1 and SED and other related interferents’ proteins such as ovalbumin (OVA) and bovine serum albumin (BSA). Both SEA-MIP and SEB-MIP sensors showed a highest sensor response to SEA and SEB, respectively, comparing to non-target analytes. Furthermore, the developed sensors were applied to detect SEA and SEB in spiked milk samples. They showed recoveries in the range of 97 to 114.2% suggesting the application of these sensors to test the SE presence in real samples. The complete data of SEA-MIP and SEB-MIP sensors for spiked milk samples are shown in Table 2.

### 4.2. Detection of Viruses

The detection of viruses is an important area of analytical research, not only because of relevance public health issues, but also due to their much smaller size than other bioanalytes. The cutting edge technologies including an enzyme linked immune sorbent assay (ELISA), polymerized chain reaction (PCR) and other sequencing methods offer very sensitive detection of viruses in complex biological samples. Nevertheless, the use of MIP-QCM sensors for label-free detection of viruses [114,115] is of considerable interest due to short analysis time and simple operation in reduced cost.

The surface stamping method has been extensively applied for imprinting different types of viruses such as tobacco mosaic virus (TMV), parapox ovis virus (PPOV), human rhinoviruses (HRV) and others. A typical virus stamp can be made by putting the virus solution on a glass/PDMS slide and allowed for sedimentation. After a certain time, this stamp is pressed over a pre-polymer surface to imprint viruses under controlled conditions and allowed to harden the layer. After washing the viruses from polymer layer, the resultant surface contains adapted cavities that can selectively accommodate the target virus. Hayden et al. [116] observed that the amount of the virus used in stamp making has a significant impact on sensor response. It was noticed that a monolayer of TMV on polyurethane surface yields a much higher sensor effect than hypomonolayer or hypermonolayer. This suggests that a virus monolayer yields optimal imprinting density and gives a better transfer of its structural details. The selectivity of the TMV-imprinted layer coated on 10 MHz QCM was tested on exposing to HRV serotype 2 virus and it was found that 8 ng/mL of TMV gives 70 Hz frequency shift, whereas 10 µg/mL of HRV results only in a 10 Hz response. The calculated selectivity factor was three orders of magnitude. A similar trend was observed when an HRV-2 imprinted layer was exposed to 960 ng/mL HRV, which yields a 1400 Hz response, while a TMV solution having even five times higher concentration had not shown any detectable signal. These results demonstrate a virus *intergroup selectivity* by MIP-QCM sensors, as they can distinguish between TMV and HRV that have different geometries and functionalities.

**Figure 5:**
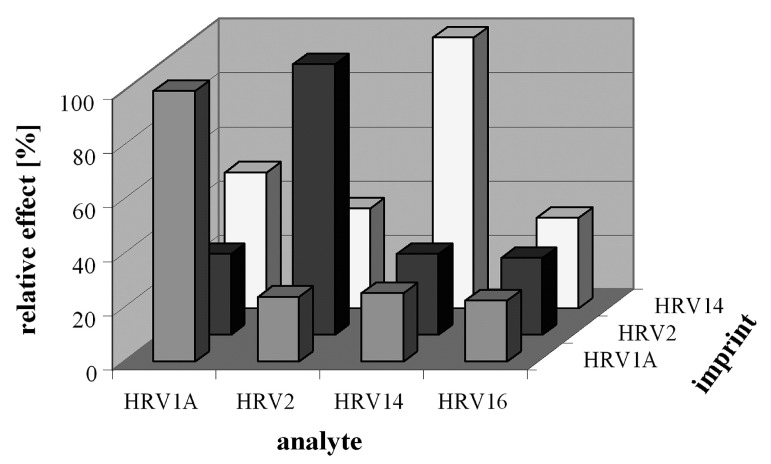
Comparison of relative sensor signals of three different MIP layers imprinted with HRV1A, HRV2 and HRV 14, respectively, is shown. When exposed to HRV1A, HRV2, HRV 14 and HRV 16, all three sensor layers showed the highest signal to virus that was used as a template for imprinting while the response for other serotypes were much lower despite of their similar geometries, reproduced with permission from [117].

In a further study, the surface stamping technique was used to achieve *intragroup selectivity* where viruses having similar geometrical sizes and surface groups can be distinguished from each other. Jenik et al. [117] imprinted different HRV serotypes i.e., HRV-1A, HRV-2 and HRV-14 on three different polyurethane layers already coated on QCM devices, respectively. These three different sensor layers were exposed to HRV-1A, HRV-2, HRV-14 and HRV-16 virus solutions and the relative sensor responses are shown in Figure 5. This graph showed a suitable *intragroup selectivity* pattern as each sensor preferably binds to the same virus that was used as a template for imprinting. For instance, an MIP sensor imprinted with HRV-1A showed the highest sensor signal for HRV-1A, whereas the response for HRV-2, HRV-14 and HRV-16 is at least three times smaller. A similar trend was observed by HRV-2 and HRV-14 sensors. Since all of these serotypes of HRV have similar sizes, therefore, simple geometrical fitting of these viruses in imprinted surface should lead to similar responses. However, the surface stamping technique allows the transfer of complete structural details of template virus thus, the resultant imprinted surface offers complementary chemical fitting to target HRV virus, which makes it possible to distinguish different HRV serotypes. In the same article, the authors observed a similar selectivity pattern when an HRV imprinted sensor was exposed to a foot–mouth disease virus (FMDV) that has a 5 nm smaller size. Although FMDV belongs to the same class of HRV i.e., picornaviruse and having even a smaller geometry would not lead it to chemically fit into HRV-imprinted cavities.

**Figure 6:**
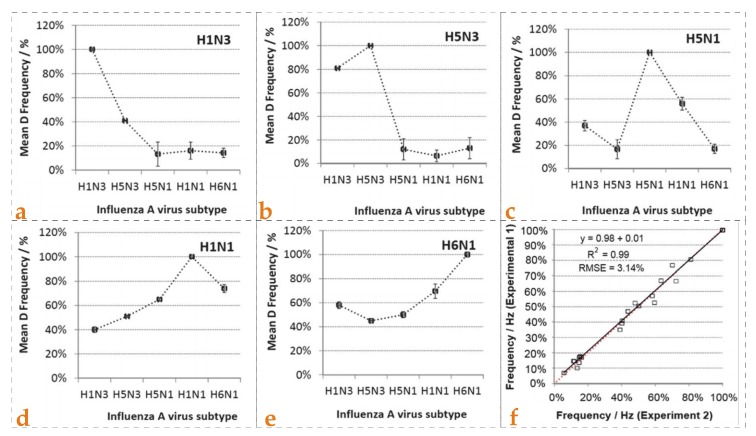
The relative sensor effects of five different sensors designed for monitoring influenza A virus sub-types are shown from (**a**–**e**). It is evident that each MIP layer showed the highest sensor response to the virus that was used as template during imprinting; (**f**) shows the reproducibility studies as a sensor signal of two MIP layers imprinted with the same virus sub-type and compared, the R^2^ value was 0.99 and standard error was found 3.1% for 50 measurements, reproduced with permission from [88].

Wangchareansak et al. [88] developed influenza A virus sensor following a surface stamping strategy and applied this system for the screening of virus sub-types. They synthesized five different MIPs for five different strains of influenza virus i.e., H5N1, H5N3, H1N1, H1N3 and H6N1, respectively. Each MIP sensor was exposed to five different solutions, each containing one virus sub-type. The data is presented in Figure 6a–e, where it is evident that each MIP sensor preferentially binds to its template virus and thus results in highest relative frequency shifts. The response to other non-target virus strains was much less in all the cases. Additionally, the reproducibility of the sensor setup was also studied by comparing the frequency shifts of two MIP sensor layers imprinted with the same template. The relative frequency shifts are shown in Figure 6f where the R^2^ value i.e., 0.99, and a standard error of 3.1% for 50 measurements was observed, thus indicating high reproducibility of developed sensor. The authors also studied the principle component analysis (PCA) model and proposed that the difference in sensor responses could be correlated with variations in hemagglutinin and neuraminidase pattern. This report demonstrates the potential of MIP-QCM sensors for selective screening of influenza virus subtypes with adequate consistency.

Tai et al. [118] explored epitope imprinting for the detection of dengue virus using a 9 MHz QCM device. They used a pentadecapeptide i.e., 15-mer peptide fragment, which is a linear epitope derived from a non-structural protein 1 (NS-1) of the dengue virus. The developed sensor showed high sensitivity and selectivity for recognizing dengue virus proteins and also can be used to detect antibodies at an MIP-NS1 interface.In a further study [119], the detection of the dengue virus in serum samples was compared by PCR, NS1 antigen ELISA, antibody-QCM and a prototype MIP-QCM sensors. Table 3 summarizes the data of these methods. The serums’ samples were diluted to 1000 fold with a PBS buffer and incubated at 95–98 °C for five minutes. In this data, it can be seen that, among 10 samples, five were identified positive and the remaining five were negative both by PCR and ELISA methods. Among five dengue virus positive samples, four of them showed frequency shifts greater than 13 Hz, while the fifth sample showed a response of 8 Hz by the MIP-QCM sensor. This suggests that the cut-off value for negative samples could be set around 6–7 Hz. On the other hand, the antibody-QCM sensor showed a much higher cut-off frequency i.e., 24–25 Hz for negative samples. Furthermore, the overall response of antibody-QCM sensor is higher, which could be due to contaminants in serum. The authors further studied that a rigid MIP-QCM sensor can be used to detect the dengue virus from reported cases within one hour and have good agreement with ELISA findings. This method demonstrates the possibility of detecting dengue virus infections at an early stage in quick time with enhanced reliability.

**Figure 7:**
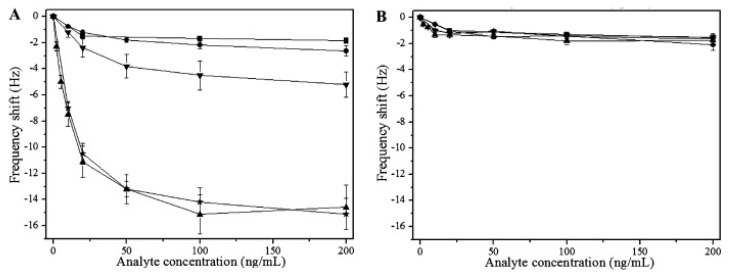
The sensor responses of imprinted (**A**) and non-imprinted (**B**) are shown towards HIV-1 *gp*41 related peptide (▴), 2M-peptide (★), 11M-peptide (▾), GA-16-NH2 (●) and BSA (■). The error bars shows the standard deviation in triplicated experiments, reproduced with permission from [120].

Lu et al. [120] developed an MIP-QCM sensor for detecting the human immunodeficiency virus type 1 (HIV-1) using epitope imprinted polydopamine as a receptor. They targeted the detection of an HIV-1 related protein i.e., glycoprotein 41 (*gp41*). A synthetic peptide fragment having 35 amino acid residues was selected as a template epitope for imprinting, as this was similar to the terminal residues of 579–613 of *gp41*. This MIP-QCM sensor can recognize template peptides as well as the related protein with adequate sensitivity. The calculated detection limit was 2 ng/mL i.e., comparable to ELISA. For selectivity evaluation, the sensor responses of imprinted and non-imprinted coating are shown in Figure 7A and 7B, respectively. They tested HIV-1 related *gp41* peptide, 2-M (two mutated amino acids peptide), 11-M (eleven mutated amino acids peptide), GA-16-NH_2_ (control peptide) and BSA. The response of the imprinted layer for the template peptide was about six times higher than BSA and control peptide; furthermore, the response of 11-M was considerably small. As the 2-M has only two mutated amino acids and has similar functionality to the template peptide, it showed a similar sensor effect. The non-imprinted channel showed an almost similar response to all the tested analytes. Moreover, the authors spiked human urine samples with HIV-1 *gp41* and tested them with the MIP-QCM sensor, which showed recoveries in the range of 86.5–94.1%, thus showing the possibility of HIV detection in real samples with suitable precision.

**Figure 8:**
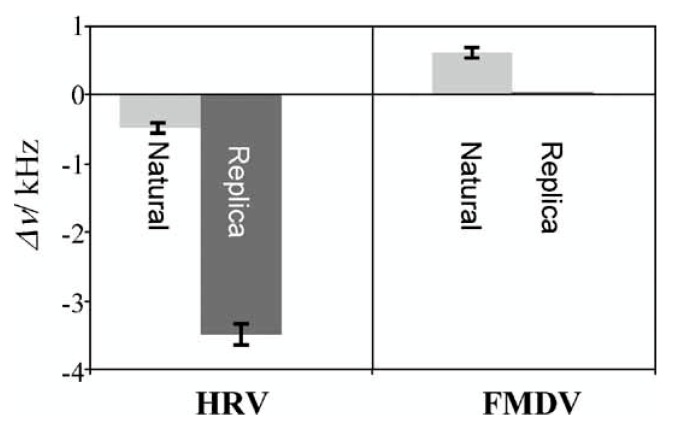
Comparison of sensor responses of natural antibodies and plastic replica of antibodies prepared by imprinting against HRV and FMDV, the replica antibodies were imprinted with HRV, reproduced with permission from [121].

Schirhagl et al. [121] synthesized natural antibodies’ imprinted nanoparticles and used them as stencil stamps that were pressed over pre-polymer layers to generate plastic replica of antibodies. The foremost advantage of using this strategy is transferring the complete biological details of antibodies (used for imprinting) into synthetic polymers. This leads to the formation of artificial copies of natural immunoglobulin (Ig). The authors compared the cross-sensitivity of biomimetic/replica antibodies with natural antibodies for detecting HRV (target) and FMDV (non-target) viruses as shown in Figure 8. The response of the replica layer was surprisingly six times higher than natural antibodies for HRV recognition. Such a high immune response by the replica layer indicates that the MIP surface offers a larger interface for target virus interaction compared to natural antibodies. On the other hand, the response to FMDV by the replica layer was negligible, whereas the natural antibodies exhibited a positive frequency shift i.e., anti-Sauerbrey effect [122] showing that the surface is not rigid. Furthermore, the response was fully reversible, which suggests that recognition is achieved through non-covalent forces. The authors also studied the sensor to sensor variation that was found to be about 10%, thus indicating the high reproducibility of this technique.

## 5. Conclusions

In this review article, we presented the exemplary applications of MIP-QCM sensors for label-free detection of bacterial and virus species as preferred micro and nano sized bioanalytes, respectively. The main imprinting strategies for their recognition and fundamental principle of QCM operation particularly in liquids are adequately discussed. From the imprinting perspective, different variables affecting the recognition performance of polymer layer are considered for optimal results. For example, we described the selection of suitable template concentration for stamp formation and length of peptide sequence for epitope imprinting. The sensor responses of different polymers for a specific target are compared to select the most suitable polymer. Moreover, imprinting in ab initio synthesized and ready-to-use polymers is also studied to evaluate their recognition properties.

In a competing environment of labeled biosensors, MIP-QCM devices offer simpler and uncomplicated recognition of target analytes without any external agent. From the above-mentioned examples, it is evident that MIP-QCM sensors are highly sensitive, selective and offer faster analysis of bioanalytes compared to other transducers having the same recognition interface. The significant feature of these sensors is their ability to precisely recognize target bacteria/viruses in complex mixtures such as human serum/urine samples. Furthermore, in various cases, these sensors can even be used for distinguishing virus sub-types, which is remarkable. In comparison to natural antibody-immobilized QCM, the MIP-QCM sensors offer comparable sensitivity and selectivity. The main driving factors for choosing MIPs over antibodies in sensor development are the straightforward synthesis, adequate stability in harsh conditions and ultimately the price of MIPs [22], which is 100-fold less than natural receptors for a particular target. In addition to this, the analysis time of MIP-QCM setup is significantly reduced compared to modern immunoassay techniques such as ELISA or PCR. Thus, the overall characteristics of MIP-QCM sensors are quite promising and indicative of their potential for cost effective and reliable detection of bioanalytes.

Despite these prominent applications of MIP-QCM sensors, molecular imprinting strategies need further in-depth research for developing more specific synthetic coatings having minimal cross-sensitivity to interferents. Over the last few years, the use of natural antibodies [123] in molecular imprinting protocols is also appearing for bioanalytes detection. The obvious advantage of using native antibodies along with synthetic systems is enhanced recognition features for detecting target bioanalytes. Nonetheless, the formation of more stable and robust sensor coatings is another aspect that needs to account for multiple measurements in corrosive medium. This is highly important to reduce layer to layer variations for larger scale production of MIP coatings as it would lead to obtaining reliable and consistent results. In view of miniaturization and multiplex measurements, the tetra-electrode QCM geometry is an appropriate example of such transducer design. By covering the four channels with appropriate MIP layers, the developed setup could be adopted for screening or differentiating between closely related bioanalytes on a single chip. Furthermore, the concept of VSA, i.e., using a single device at multiple harmonics, is also an interesting strategy for precise molecular weight estimation studies.

In summary, more detailed and insightful research towards imprinting strategies for enhanced molecular recognition and an improved QCM sensor array design would lead to the development of efficient MIP-QCM platform for offering rapid analysis with suitable accuracy at a reduced cost. The development of such a label-free sensor array could be used for diverse applications including biotechnology, clinical diagnostics, and detection of infectious or pathogenic species in food or drinking samples and other related bioassays. 

## Figures and Tables

**Table 1:** Comparison of SPR and QCM sensors data for *E. coli*, the tested concentration range for SPR was 0.5–4.0 McFarland and for QCM was 0.5–3.0 McFarland, adapted from [111].

Sensor Type	Flow Rate (μL/min)	Response Time (s)	Time to Reach Stable Signal (min)	Analysis Time for 1 Cycle (min)	Langmuir Isotherm (R^2^)	Limit of Detection (CFU/mL)
**SPR**	150	113	15	20	0.9461	1.54 × 10^6^
**QCM**	350	56	5	7	0.9931	3.72 × 10^5^

**Table 2:** The observed concentrations and % recovery values of SEA-MIP and SEB-MIP sensors for spiked milk samples, —^a^ indicates not detectable concentration, reproduced with permission from [112].

Sample (ng/mL)	SEA-MIP QCM Sensor		SEB-MIP QCM Sensor	
	Found (X^-^ ± S)	Recovery (%)	Found (X^-^ ± S)	Recover (%)
Blank	—^a^	—	—^a^	—
5	4.85 ± 0.92	97.00	5.71 ± 0.35	114.20
50	52.06 ± 3.66	104.12	46.71 ± 2.13	93.42
100	97.02 ± 1.46	97.02	109.02 ± 3.25	109.02

**Table 3:** Comparison of MIP-QCM sensor data with antibody-QCM, PCR and *NS1* antigen ELISA results for detection of dengue virus infections in clinical samples. ND indicates not detectable, reproduced with permission from [119].

Specimen Code, n	Sex	Age (Year)	Sampling Time (Day)	PCR Result	*NS1* Antigen ELISA, A (SD)	Antibody-QCM (Hz)	MIP-QCM (Hz)
25114	M	41	1	DEN-2	1.68 (0.13)	37	21
25230	M	29	4	DEN-2	1.87 (0.19)	40	29
25339	M	60	4	DEN-2	1.64 (0.11)	48	14
25348	F	17	4	DEN-2	1.72 (0.09)	32	17
25433	F	14	4	DEN-2	1.02 (0.10)	26	8
26093	M	52	19	ND	Negative	21	5
26094	F	17	21	ND	Negative	17	0
26096	F	9	18	ND	Negative	17	3
26134	M	49	13	ND	Negative	23	6
26143	F	25	1	Negative	Negative	9	5
